# Recommended by Physicians

**Published:** 1863-07

**Authors:** 


					﻿“RECOMMENDED BY PHYSICIANS.”
The U. S. Recruiting Officer in Buffalo, informs us that the irregular
physician, who was at one time employed by him in examining recruits,
was recommended to him as a “suitable and capable person for such duty,
by intelligent, regular physicians, and that these physicians regard O.ur arth
Page(s) missing
The report of Dr. A, K. Gardner, of New York, regarding the use and
ahuse of pessaries, the reading of which was yesterday postponed until this
morning, was called up, as next in order, and on motion, the reading of it
postponed until next year.
The Committee appointed to prepare suitable resolutions appropriate to
the loss of the Association by death of its late President, the late Dr. Eli
Ives, of Connecticut, made their report, which, after a slight amendment,
was adopted.
The Committee on Voluntary Communications presented an abstract of
a paper by Dr. Andrews, of Chicago, on “Diatheses—Their Surgical Rela-
tions, ” which was read by the author. Approved, and referred to the Com-
mittee of Publication.
The meeting then adjourned until afternoon.
AFTERXOOS SESSIOS.
According to a resolution passed this morning. Dr. D. J. Macgowan, of
New York, from China and Japan, was invited to address the Association.
He explained to the meeting the professional bearings of his proposed
scaentifc and rndnsitrial expedition of the unknown parts of Eastern Am.
Dr. C. C. Cox, from the Committoe on Medical Education, read an able
saentifie paper on the subject, reviewing the past history of the profession in
this respect, and the 'absence of proper attention to the subject. Many vaL
nablle mgsjjaffittiksBs as to needed impftfoanerita, were also made. After the
rendering of tthferepsnt, the Committtoe subndttod the Mowing resolutions,
whfeh, riffler dnsemremm, were adapted:
JifewfeWThrit a thorough jjaeffimmany education in English, Latin,
and physics amnafitiBtos am etoemtiall pre-requisite to the adnm-
skw of a asttmdantt off mriSnsue into the ofc® of a medical preceptor, or as a
umtoriiaribnit of a n®qpsjftalblfe mediim! ©affllege.
^fsadbuxB,Thrit the advammnmntt of msdieal edumetiom demands a more
extefimdtd awfl symmsttniicEl ©rawffle <df'imBstawltom inn ttlhie ©efflteges, and a more
ttltaaiough and impanttM exarnunrifeui hmr the degree of daatfor of medfeme
tfium rit p®gflrit pneraflL
TIhaft MefficuD Jhm^pnuidaaiwand Hygiene areBtgfoly impmtamt
hnanaftn® ®ff Me&ail Seuheskj, dfiaarwihg the onefM ©snrideuattiom of all medical
taadbsisanid gahreM
TEiftuflhmil—srawrorimR flair mnmffiwiril	—State), dnrifimtt and
©sunHy,—anayn^wittaiift qanri^rnmaB to the adunntenmnit and promotion of
sofanse, aniil an® tthawtoi® highly nffimunmauiled By this Body, as vtaBnaBfe
Itewsrs ihttffloffi' oara® of	nsBucrifimn.
Waft (Chnnmittoe ajppanted to undto a. nrtproitt upona the reeartt onder of the
S&UKgHHii (GtnisrdI, jpiuhiiiitihg the uh® off iniHKfuauiiiifc and tauittaihred anthnooiiy
By tdiie anmy fSuugghdl	imafe ;a rmigjffliuittw repent; tthnough Du. JLamon^
Qff©mijiniHtij, anrilam ®rttiixdly	mMOMnftty repsutt By Dm. Wood-
Mraputfij, off Uuiifi iuua. TEhe ffimrmwr attorney ffiwunffld the uh® of these remedial
agjfflits im the ?auny„ amll the llitttar ats frihxaii^ly diiKBauihmaauiei theiir uh®
tdffflye. Bhuh ixsqpoitt tow Ibuilhsll up) By resBdhtfhiDB niigjidlly unidonsiig tfhe
IhngpngpB off tftie haffinmfy, afttoar tot mmnnitafl dfamitoran.
				

